# Work-related musculoskeletal disorders : A survey of physical therapists in Izmir-Turkey

**DOI:** 10.1186/1471-2474-5-27

**Published:** 2004-08-18

**Authors:** Yesim Salik, Ayse Özcan

**Affiliations:** 1 School of Physical Therapy and Rehabilitation, Dokuz Eylül University, PO Box 35340 Inciralti, Izmir, Turkey

## Abstract

**Background:**

This study was planned to collect data about causes, prevalence and responses to work-related musculoskeletal disorders reported by physiotherapists employed in Izmir, Turkey.

**Method:**

A two-page survey with closed ended questions was used as the data collected method. This survey was distributed to 205 physiotherapists working in Izmir, Turkey, and 120 physiotherapists answered. Questions included occupational history of physiotherapists and musculoskeletal symptoms, special areas, tasks, job-related risk factors, injury prevention strategies, and responses to injury.

**Results:**

Eighty-five percent of the physiotherapists have had a musculoskeletal injury once or more in their lifetime. Injuries have been occurred mostly in low back (26 %), hand-wrist (18 %), shoulders (14 %) and neck (12 %). The highest risk factor in causing the injury was transferring the patient at 15%. Sixty-nine percent of physiotherapists visited a physician for their injury and sixty-seven percent of the respondents indicated that they had not limited their patient contact time as a result to their injury

**Conclusions:**

According to the results of this study, the rate of musculoskeletal disorders in physiotherapists in Izmir-Turkey has been found to be high due to their profession. Respondents felt that a change in work habits was required in order to decrease the risk of another injury.

## Background

A work-related musculoskeletal disorder (WRMD) is defined as a musculoskeletal injury that results from a work-related event. This may result in lost work time, work restriction, or transfer to another job [1-4]. These types of injuries are common among physiotherapists [1,2,5-9]. This group has a moderately high prevalence of occupational low-back pain [2,9-12]. Physical therapists routinely perform manual therapy, such as soft-tissue mobilization, which means that the upper limb is also exposed to risk factors associated with musculoskeletal and neurovascular disorders [1,2,6-8,13,14]. In addition, these professionals routinely perform activities that involve transferring a patient (from exercise mat to chair, to parallel bar etc), assisting with activities on the exercise mat, and lifting and using cumbersome equipment [1,2,6-15]. These work tasks put therapists at risk for both acute and cumulative musculoskeletal pain.

Understanding the issues related to musculoskeletal disorders in physical therapists requires some awareness of the context in which these professionals work. In Turkey, physiotherapists cannot treat patients directly. According to law, direct accesses to physiotherapists are not possible [16]. Further, physiotherapists are not allowed to plan patients' physiotherapy programs; this is done by a physiatrist. Currently, efforts are underway to enact legal amendments that will permit a doctor to direct a patient to a physiotherapist, with the physiotherapist creating the physiotherapy program him- or herself.

The purpose of this study was to investigate the prevalence and features of WRMDs in a group of Turkish physiotherapists working in various capacities in a large Turkish city, and to compare the findings to reports from other countries.

## Methods

The study was approved by the Ethics Committee of Dokuz Eylül University. The research population was selected physiotherapists who were employed in a broad spectrum of practice settings in İzmir, Turkey. All data were collected by a mailed questionnaire (Appendix 1 [see Additional file 1]) that consisted of 17 close-ended questions. Questionnaires were distributed to all physiotherapists (205 total, 157 females and 48 males) who were registered members of the Izmir Branch of the Turkish Physiotherapy Association. Each member was asked to complete the self-administered questionnaire if they had more than 2 years of experience in practice. One hundred and twenty questionnaires were returned and analyzed (total response rate 59%, for women 59%, for men 58%).

The questionnaire was based on another published survey [1,6] and simply adapted and translated for the Turkish context. The questionnaire was composed of two parts, personal and occupational. The personal portion asked about general characteristics, including sex, age, weight, and height. The occupational portion inquired about years of experience, work setting, and number of hours of contact with patients per week. This section also asked whether the subject had experienced any WRMDs. If the answer was yes, the person was asked to state the type of injury, the body part affected, specific activities caused on occupational injury, the work setting in which the injury occurred, whether the injury was reported or a physician was consulted, and what sort of treatment was applied. They were also asked whether they lost work time as a result of the injury, what activities caused symptoms to recur, and whether the injury had caused the respondent to alter his or her work habits, reduce hours with patients, or change employment settings.

Data were analyzed using SPSS 10.0 for Windows. Results for the general information items were expressed as mean ± standard deviation, and results for items in the occupational portion were expressed as percentages. χ^2 ^were used to analyze influence personal characteristics (sex, age, number of years in physiotherapy practice, number of hours per week in direct patient care) to WRMDs.

## Results

The 120 respondents included 92 females and 28 males of mean age 30.4 ± 6.9 years (range, 22–55 years). Total response rate was 59%, for women 59%, for men 58%. General information about the group is given in Table 1. The questionnaire answers indicated that the respondents spent an average of 38.8 ± 9.2 hours per week in direct patient care, and had 8.0 ± 6.0 years of work experience on average. Eighty-six of the physiotherapists worked in general hospitals, 24 worked at hydrotherapy centers, and 10 worked at pediatric rehabilitation centers.

Frequency of WRMDs was not correlated with sex (χ^2^=.234, P=.629), age (χ^2^=.383, P=.536), number of years in physiotherapy practice (χ^2^=.067, P=.795), or number of hours per week in direct patient care (χ^2^=.151, P=.698) (Table 2).

One hundred and two respondents (85%) reported that they had experienced work-related musculoskeletal pain or discomfort at some time in their occupational lives. Seventy-eight (65%) reported that they had sustained more than one WRMD.

The lower back was the body part with the highest frequency of occupational injury (26%), and wrist-hand (18%), shoulder (14%), and neck (12%) were other sites frequently affected (Table 3). The main types of injury reported were tendinitis (21%), vertebral disk problems (16%), muscle strain (16%), ligament sprain (16%), degeneration (15%), synovitis (6%), tear (2%), dislocation (1%), fracture (1%) and other (6%). The factors that most frequently led to WRMD were transferring patients (15%), performing repetitive tasks (14%), and lifting (14%) (Table 4).

The respondents who had experienced a WRMD indicated that lifting (18%), maintaining a position for prolonged period of time (17%), performing repetitive tasks (16%), and transferring patients (16%) were the activities that most often exacerbated their symptoms during clinical practice (Table 5). Improvements in body mechanics (21%), avoiding lifting (16%), and changing working positions frequently (14%) were the top three changes that the injured respondents said they made in their working habits (Table 6).

Sixty-nine percent of the respondents with WRMDs had visited a physician for the problem, and 46% of whom stated that they had officially reported the injury to their employer. The respondents who had suffered WRMDs reported they used their own occupational knowledge, rest, medications, and exercise to treat the problem (Table 7). Sixty-seven percent of the respondents who had had WRMDs indicated that they had not permanently reduced their patient-contact time as a result of their injury, and 82% said they did not limit their areas of practice after sustaining the injury. Most of the physiotherapists (63%) who had had WRMDs indicated that they would not consider a job change because of their injury or due to the risk of sustaining another injury.

## Discussion

Musculoskeletal system problems connected to occupational conditions are common among health care workers. The costs of these are substantial, both in terms of money and in terms of work time lost [1,7,9,17,18]. Research has shown that musculoskeletal problems are particularly common in health care workers who are in direct contact with patients [1,7,8,17,18]. Physiotherapists have a high prevalence of WRMDs [1,7,8,15].

The results from studies on WRMDs in physiotherapists have generally been similar, though some have differed according to country. Such variations are linked to level of development, the status of the profession of physiotherapy in a given country, psychosocial, and epidemiological factors [2,5,8,11,13,14]. In Turkey the law bars physiotherapists from providing primary care; each patient must be referred. Considering the differences in physiotherapy practice among countries and regions, we felt it would be valuable to investigate the prevalence and features of WRMDs in a group of Turkish physiotherapists working in various capacities, and to compare to findings in other countries.

In this study, we collected demographic and WRMD data from 120 physiotherapists in Izmir, Turkey, and analyzed rates of injury, risk factors, injury types and sites, and post-injury management. We asked to complete the self-administered questionnaire if they had more than 2 years of experience in practice. Thus, response rate of questionnaire was low (58.5 %) in our study. The survey answers revealed that 85% of the respondents had experienced WRMDs. Cromie [8] reported that younger physiotherapists have a higher prevalence of musculoskeletal problems related to occupational conditions. Rugelj [9] investigated low-back pain in physiotherapists, and found an incidence of 66% in subjects between the ages of 20 and 40 years [1]. In line with this, a study of Australian physiotherapy students by Nyland [2] revealed that the 20- to 21-year age group had the highest frequency of low-back pain. The average age of the physiotherapists in our study was 30.4 years. This mean age corresponds with other findings in the literature, and confirms that physiotherapists tend to experience WRMDs at young age. Such injuries in younger physiotherapists may be associated with lack of professional experience, and the lower knowledge and skill levels people tend to have in the early years of this career.

Concerning sites of musculoskeletal injury during professional activities, the highest incidence is in the low-back region. Biomechanical studies have shown that physical loading factors, such as body flexion, rotation and weight loading, play a role in this [18]. In a study that covered 25% of all physiotherapists working in Australia (824 total studied), Cromie [8] found that the rate of work-related low-back pain was 48%. Other authors have revealed various rates of this problem in physiotherapists: Bork [7] 45%, Holder [1] 62%, Molumphy [10] 29%, Scholey and Hair [12] 38%, Mierzejewski [11] 49.2%, Rugelj [9] 73.7%, and Nyland [2] 69%. Our survey of Turkish physiotherapists revealed a 25.5% incidence of low-back pain. Interestingly, this figure is lower than most other rates reported in the literature. In our study, when we categorized physiotherapists according to practice setting, low-back pain was the most common WRMD in all subgroups.

Studies of WRMDs in health care of professionals have identified the lower back as the most commonly involved area of the body, followed by neck and upper extremities [3,13,19-21]. Investigations of physiotherapists have revealed similar results. Bork [7] and Holder and et al. [1] listed the regions most commonly involved musculoskeletal injuries as the lower back, hand-wrist, and neck, respectively among physiotherapists. On analyzing the different body parts injured in our subjects, we found the highest frequency of injuries in the lower back, followed by the hand-wrist (18.2%), shoulder (14.4%) and neck (11.8%).

According to the literature the work-related activities that most commonly lead to injury in health professionals are lifting heavy equipment and patients, transferring patient, maintaining the same posture for a long period, manual therapy practices, responding to patients' sudden movements, and repeated movements [1,8,17,18,22-24]. Bork [7] identified the main causes of WRMDs in physiotherapists as staying in the same position for along time and continuing to work when tired; Molumphy [10] emphasized lifting and leaning downwards with sudden maximal effort; and West [13] highlighted maintaining the same posture for along time, manual therapy, repeated movements, and increased work load. In our survey, the main causes of WRMDs were patient transfer, repeated movements, lifting heavy equipment, patients and working when too physically tired.

Holder et al. [1] identified three activities that aggravate the symptoms of former WRMDs in physiotherapists and assistant physiotherapists as staying in the same position for a long period, lifting and patient transfer. In our study, the respondents to our questionnaire noted lifting, staying in the same position for a long time, patient transfers, and repeated movements.

In Turkey, most physiotherapists work in general hospitals. In these facilities, the daily treated patient number is too high, and this number far exceeds the number of physiotherapists on staff. In addition, the majority of patients in these hospitals are seriously ill [25].

People who suffer injuries on the job may be treated with medication, rest and exercise. Physiotherapists have fundamental knowledge about ergonomics and biomechanics, and using this knowledge may vary depending on professional knowledge and skills. In Turkey, physiotherapists are trained in ergonomic working principles at the undergraduate level. However, in many working environments the equipment used (treatment table, chair, *etc*.) is not ergonomic, and consequently the physiotherapist cannot follow ergonomic principles while doing his or her job.

A study conducted by Cromie [6] in 2002 examined whether physiotherapists use their own knowledge to prevent WRMDs. The author found that this was true for most of the physiotherapists investigated. In our survey, of the physiotherapists who had suffered WRMDs, 27.6% said they used their professional knowledge and 26% said they used rest to manage the injury.

Previous research has shown that physiotherapists who have suffered a WRMD tend to change their professional attitudes to avoid additional injuries. Holder *et al. *[1] found that 79% of physiotherapists and 81% of assistant physiotherapists who had suffered injuries on the job changed their professional attitudes to avoid other WRMDs. It has been reported that the most common strategies used by physiotherapists to avoid WRMDs are correction of body mechanics, and frequent postural changes. The respondents to our questionnaire said that, after suffering a WRMD, they paid more attention to correcting body mechanics, avoided lifting heavy equipment or patients, changed position frequently, and got other personnel to help them with tasks that involved lifting.

Mierzejewski [11] reported that 13.7% of physiotherapists in Edmonton, gave up their career after suffering work-related low-back pain, and that 35.3% continued to work after this problem. Cromie [8] found that 17.7% of physiotherapists changed their field of practice in connection with WRMDs. Of the respondents in our Turkish survey who had suffered WRMDs, 67% did not change their field of practice after the injury, 82% did not restrict their time with patients, and 64% said they would not consider changing their field or department of practice due to their WRMD. This means that 33% did change field of practice after a WRMD, which is 1.9 times the rate of those who changed field in Cromie's study. As noted above, most physiotherapists in Turkey work in general hospitals, and it is not usually possible for such physiotherapists to change their field of work.

Some professions have very high WRMD prevalence, and this has led to more intensive research in recent years. These types of injury have major consequences for society, workers, employers, and the insurance sector due to loss of labor force, long-term disability and delay in returning to work, decreased productivity, and psychological effects on employees. Therefore, minimizing and preventing WRMDs has significant potential social and economic benefits. It is important to invest in studies aimed at reducing these types of injuries in physiotherapists.

Our study has three main limitations. A physiotherapist's working time in a day is not long, but he or she treats a very large number of patients during this time. Our survey would have been more informative if we collected data for the number of daily treated patients. We also did not inquire about the physiotherapists' activity levels. This would have been valuable information, as sports and recreational activity may affect WRMD frequency. Another limitation of our study was comparing various areas of practice and techniques used by physiotherapists and investigating their relationship to WRMDs.

## Conclusions

Our survey reveals that the WRMDs in physiotherapists in Turkey are similar to rates reported in other countries. Physiotherapists in our country suffer similar work-related injuries as their counterparts elsewhere, despite different legal working conditions and cultural differences. This study provides data related to WRMDs in physiotherapists in Turkey. Further studies can be very useful if it research prevalence of WRMDs in physiotherapists who have employed different working conditions.

## Competing interests

None declared.

## Authors' Contributions

YS participated in the design, managed the data collection and performed statistical analysis.

AO participated in designed the study protocol, managed the coordination.

All authors read and approved the final manuscript.

## Pre-publication history

The pre-publication history for this paper can be accessed here:



## Supplementary Material

Additional File 1 Table 1, Table 2. Questionnaire on occupational injuries in physical therapists. Turkish version of questionnaire on occupational injuries in physical therapists Click here for file

## Figures and Tables

**Table 1:** Descriptive information of physical therapist (PT) respondents to questionnaire

		**Female (n = 92)**	**Male (n = 28)**	**All**
Age (yrs)	Mean (SD)	30.3 (7.5)	30.8 (4.4)	30.4 (6.9)
	Range	22 – 55	25 – 38	22 – 55
Height (m)	Mean (SD)	1.63 (6.21)	1.76 (7.3)	1.67 (8.41)
	Range	1.32 – 1.78	1.65 – 1.90	1.32 – 1.90
Weight (kg)	Mean (SD)	57.9 (6.4)	78.1 (13.1)	62.6 (12.0)
	Range	45 – 82	53 – 100	45 – 100
Years as a PT	Mean (SD)	8.2 (6.7)	7.9 (3.6)	8.0 (6.0)
	Range	2 – 29	2 – 14	2 – 29
Hours per week in direct patient care	Mean (SD)	38.5 (9.0)	40.0 (9.8)	38.8 (9.2)
	Range	8 – 60	5 – 60	5 – 60

**Table 2:** Distributions of the physical therapists (PT) with WRMDs according to sex, age, years as a pt, and hours per week in direct patient care

		WRMD (n)	%
Sex	Female	79	85.9
	*Male*	23	82.1
Age (yrs)	<*30*	59	86.8
	≥30	43	82.7
Years as a PT	<*8*	60	85.7
	≥8	42	84.0
Hours per week in direct patient care	<*38*	24	82.8
	≥*38*	78	85.7

**Table 3:** The frequencies of WRMDs at body part affected in different practice settings.

	Orthopedic Rehabilitation %	General Physical Therapy %	Neurological Rehabilitation %	Cardiopulmonary Rehabilitation %
**Neck **n:30 12 %	9.1	10.9	15.1	6.7
**Shoulder **n:39 14 %	11.4	16.9	15.1	16.7
**Upper Back **n:15 9 %	6.8	5.0	5.8	6.6
**Elbow **n:22 8 %	13.6	7.9	8.1	3.3
**Lower Back **n:72 26 %	29.5	25.7	28.0	30.0
**Wrist/Hand **n:48 18 %	18.2	22.8	12.8	16.7
**Hip/Thigh **n:6 2 %	2.3	3.0	2.3	0.0
**Knee **n:20 8 %	6.8	5.9	9.3	10.0
**Ankle/Foot **n:9 3 %	2.3	2.0	3.5	10.0

**Table 4:** Proportions of respondents who reported that specific activities caused on occupational injury

**Activity that caused injury**	**PT (%)**
Transferring a patient	14.5
Performing repetitive tasks	13.9
Lifting heavy equipment or patients	13.6
Working when physically fatigued	12.0
Bending/twisting	11.1
Performing manual therapy	9.0
Maintaining a position for a prolonged period of the time	9.2
Responding to an unanticipated or sudden movement a by patient	5.9
Working in an awkward or cramped position	5.0
Slipping. tripping. falling	2.7
Applying modalities	1.5

**Table 5:** Proportions of respondents who reported that job activities caused their symptoms to recur

**Exacerbating activity**	**PT (%)**
Lifting heavy equipment or patients	18.1
Maintaining a position for a prolonged period of time	16.6
Transferring a patient	15.5
Performing repetitive tasks	15.8
Performing manual therapy	10.8
Squatting	5.0
Working in an awkward or cramped position	4.3
Climbing stairs	3.9
Reaching/working or cramped positions	3.5
Walking	3.5
Performing overhead activities	1.9

**Table 6:** Proportions of respondents who reported altering work habits as a result of experiencing an occupational musculoskeletal injury

**Altered work habits**	**PT (%)**
Use improved body mechanics	20.5
Avoid lifting	16.4
Change working position frequently	13.7
Increase use of other personnel	10.5
Decrease manual therapy	6.9
Increase use of mechanical aids	7.3
Increase administrative time. decrease patient care time	7.3
Encourage patient responsibility for carrying out treatment	6.4
Stop working when hurt or when symptoms occur	5.5
Change work schedule	3.2
Take more rest breaks or pauses during the workday	2.3

**Table 7:** Types of treatments and response to musculoskeletal injury of respondents

		**n**	**%**
**Treatment**	Use of occupational knowledge	70	27.5
	Rest	66	26.0
	Medication	65	25.6
	Exercise	50	19.7
	Surgery	3	1.2
**Response to injury**	Visiting a physician	70	68.6
	Reporting officially	45	44.1

## References

[B1] Holder NI, Clark JM, DiBlasio JM, DiBlasio JM, Hughes CL, Schrpf JW, Harding L, Shepard KF (1999). Cause, prevelance, and response to occupational musculoskeletal injuries reported by physical therapists and physical therapist assistants. Phys Ther.

[B2] Nyland LJ, Grimmer KA (2003). Is undergraduate physiotherapy study a risk factor for low back pain? A prevelance study of LBP in physiotherapy students. BMC Musculoskeletal Disor.

[B3] Aptel M, Aublet-Cuvelier A, Cnockaert JC (2002). Work related musculoskeletal disorders of the upper limb. Joint Bone Spine.

[B4] Kilbom A (1998). Editorial / Prevention of work-related musculoskeletal disorders in the workplace. Int J Ind Ergon.

[B5] Cromie JE, Robertson VJ, Best MO (2002). Occupational health in physiotherapy: general health and reproductive outcomes. Aust J Physiother.

[B6] Cromie JE, Robertson VJ, Best MO (2002). Work related musculoskeletal disorders and the culture of physical therapy. Phys Ther.

[B7] Bork BE, Cook TM, Rosecrane JC, Engelhardt KA, Thomason MEJ, Wauford IJ, Worly RK (1996). Work related musculoskeletal disorders among physical therapists. Phys Ther.

[B8] Cromie JE, Robertson VJ, Best MO (2000). Work related musculoskeletal disorders in physical therapists: prevelance, severity, risks and responses. Phys Ther.

[B9] Rugelj D (2003). Low back pain and other work-related musculoskeletal problems among physiotherapists. Appl Ergon.

[B10] Molumphy M, Unger B, Jensen GM, Lopopolo RB (1985). Incidence of work related musculoskeletal low back pain in physical therapists [abstract]. Phys Ther.

[B11] Mierzejewski M, Kumar S (1997). Prevelance of low back pain among physical therapists in Edmonton, Canada [abstract]. Disabil Rehabil.

[B12] Scholey M, Hair M (1989). Back pain in physiotherapists involved in back care education [abstract]. Ergonomics.

[B13] West DJ, Gardner D (2001). Occupational injuries of physioterapists in North and Central Queensland [abstract]. Aust J Physiother.

[B14] Caragianis S (2002). The prevelance of occupational injuries among hand therapists in Australia and New Zealend [abstract]. J Hand Ther.

[B15] Cromie JE, Robertson VJ, Best MO (2001). Occupational health and safety in physioterapy: guidelines for practice [abstract]. Aust J Physiother.

[B16] (1923). 1219 The Act on the Practice Medicine.

[B17] Evanoff BA, Bohr PC, Wolf LD (1999). Effects of a participatory ergonomics team among hospital orderlies. Am J Ind Med.

[B18] Galinsky T, Waters T, Malit B (2001). Overexertion injuries home health care workers and the need for ergonomics [abstract]. Home Health Care Serv Q.

[B19] Hoogendoorn WE, Bongers PM, Wet de HCW, Ariens GAM, van Machelen, Bouter LM (2002). High physical work load and low job satisfaction increase the of sickness absence due to low back pain: results of a prospective cohort study. Occup Environ Med.

[B20] Buckle PW, Devereux JJ (2002). The nature work-related neck and upper limb musculoskeletal disorders. Appl Ergon.

[B21] Silverstein B, Viikari-Jutura E, Kalat J (2002). Use of a prevention index to identfy industries at high risk for work-related musculoskeletal disorders of the neck, back and upper extremity in Washington state, 1990–1998 [abstract]. Am J Ind Med.

[B22] Jelcic A, Culjak M, Horvacic B (1993). Low back pain in health personnel [abstract]. Reumatizam.

[B23] Michalak-Turcotte C (2000). Controlling dental hygiene work-related musculoskeletal disorders: the ergonomic process [abstract]. J Dent Hyg.

[B24] Fish DR, Morris-Allen DM (1998). Musculoskeletal disorders in dentists [abstract]. N Y State Dent J.

[B25] The Republic of Turkey Ministry of Health, 'Health Statistics'.

