# Supplementary material for: Work-related musculoskeletal disorders : A survey of physical therapists in Izmir-Turkey
Source: BMC Musculoskelet Disord. 2004 Aug 18;5:27. doi: 10.1186/1471-2474-5-27 (PMC516038; doi:10.1186/1471-2474-5-27)
Supplement: Additional File 1 — Table 1, Table 2. Questionnaire on occupational injuries in physical therapists. Turkish version of questionnaire on occupational injuries in physical therapists [file 1471-2474-5-27-S1.doc]

**Table 1. Questionnaire on occupational injuries in physical therapists**

**Section A:** Personal Portion

        Age: Sex:

        Height:.........cm Weight:.........kg

**Section B:** Occupational Portion

 Years of experience ----------------years

      In what are do you currently practice?

Orthopedic Rehabilitation ……………..

### General Physical Therapy……………...

Neurological Rehabilitation…………....

Cardiopulmonary Rehabilitation……….

     Hours of the contact with patients? .........hours.

      Have you sustained any musculoskeletal injuries due to your work within last 2 years?

Yes. one only --------- Yes. more than one ---------- No------------

      What body part(s) was (were) affected. Please check all that apply

...........Neck ..........Shoulder ..............Hip

...........Upper Back (Thoracic) ............Elbow ..............Knee

...........Wrist/Hand ........Ankle/Foot

...........Lower Back (Lumbar/Sacral)

      What type of injury was it? Please check all that apply.

...........Degeneration .........Synovitis .............Fracture

..........Ligament Sprain .........Vertebral Disk Problem

..........Dislocation .........MuscleStrain

..........Tear .........Neuropathy

..........Tendinitis .........Other

      What activity were you doing when you were injured?

...........Applying Modalities ...........Performing Repetitive Tasks

...........Bending/ twisting ...........Transferring a Patient

...........Lifting heavy equipment or patients

...........Slipping-Tripping-Falling

...........Maintaining a Position for a Prolonged Period of Time

...........Performing Manual Therapy Techniques

...........Working in an Awkward or Cramped Position

...........Responding To an Unanticipated or Sudden Movement by a Patient

...........Working When Physically Fatigued

...........Other

      In what type of setting did the injury occur?

..........University Hospital

..........Public Hospital

..........Pediatric Rehabilitation Center

..........Patient’s Home (Home Care)

..........Private Physical Therapy Office

..........Other

      Did you officially report the injury? .......... Yes ........... No

      Did you see a physician for the injury? ...........Yes ........... No

      Did you lose a half day or more of work as a result of the injury?

........... Yes ........... No

      What kind of treatment was applied?

..........Surgical ..........Medical

..........Rest ..........Exercises

..........Personal knowledge of physical therapy (postural adaptation. ergonomics…)

      Since your injury. have your symptoms been exacerbated by clinical practice?

........... Yes ........... No

*If Yes*. what activities cause your symptoms to recur? Please check all that apply.

---------Bending or Twisting

---------Lifting

---------Maintaining a Position for a Prolonged Period

---------Performing Manual Therapy Techniques

---------Performing Repetitive Tasks

---------Transferring a Patient

---------Performing Overhead Activities

---------Reaching

---------Climbing Stairs

---------Squatting

---------Walking

---------Working in an Awkward or Cramped Position

---------Other

      Has the injury caused you to alter your work habits?

       ........... Yes ........... No

*If Yes*. what do you do differently? Please check all that apply.

---------Avoid Lifting

---------Change Working Position Frequently

---------Change Work Schedule (Overtime. Irregular Shifts. Length of Workday)

---------Decrease Manual Techniques

---------Encourage Patient Responsibility for Carrying Out Treatment

---------Increase Use of Mechanical Aids

--------- Increase Administrative Time; Decrease Patient Care Time

--------- Increase Use of Other Personnel

---------Stop Working When Hurt or When Symptoms Occur

---------Take More Rest Breaks or Pauses during the Workday

---------Use Improved Body Mechanics

      Have you limited your patient contact time as a result of the injury?

.........Yes ..........No

      Have you limited your area of practice to avoid sustaining another injury? .........Yes .........No

 Are you considering changing jobs because of this injury or because you fear another injury?

...........Yes ........... No

Table 2. Turkish version of questionnaire on occupational injuries in physical therapists

**Bölüm A:** Kişisel Bilgiler

- Yaş: Cinsiyet:
- Boy: Vücut ağırlığı:

**Bölüm B:** Mesleki Bilgiler

- Mesleğinizde kaçıncı yılınız? ----------yıl
- Çalıştığınız departman:
- Genel olarak haftada kaç gün hastalarla direkt kontakt halindesiniz? .........saat.
- Son iki yıldır işinizde kas-iskelet sistemi problemi yaşadınız mı?

Evet, bir kere--------- Evet, birden fazla---------- Hayır------------

- Hangi vücut bölümünüz yaralanmaya maruz kaldı? (Birden fazla seçenek olabilir)

...........Boyun ...........Omuz ..............Kalça

...........Torasik bölge ............Dirsek ..............Diz

...........Lumbal/sakral bölge ...........El/elbileği ..............Ayak/ayak bileği

- Bu ne tür bir yaralanmaydı? (Birden fazla seçenek olabilir.)

...........Dejenerasyon .........Synovit ............. Fraktür

..........Ligament strain .........Vertebral disk hasarı

..........Dislokasyon .........Kas strain ...........Yırtılma(kopma)

..........Nöropati .........Tendinit ............Diğer

- Yaralanma ne çeşit aktivite/aktiviteler esnasında meydana geldi?

...........Modalite kullanma ...........Tekrarlı hareket esnasında

...........Eğilme/ bükülme ............Hasta transferi

...........Kaldırma ...........Kayma-düşme-takılma-sendeleme

...........Uzun bir süre aynı pozisyonda kalma

...........Manuel terapi teknikleri uygulama esnasında

...........Uygun olmayan veya dar alanda çalışma esnasında

...........Bir hastanın ani hareketi /beklenmedik bir cevaba karşı

...........Fiziksel olarak aşırı yorgun iken çalışmaya devam etme esnasında

...........Diğer

- Yaralanma nerede meydana geldi?
   ..........Üniversite

...........Özel fizyoterapi merkezi

...........Hastane

...........Pediatrik nöroloji rehabilitasyon merkezi (CP merkezi)

.......... Hastanın evi

...........Diğer

- Yaralanmanızı resmen raporladınız mı? ........... Evet ........... Hayır
- Yaralanma sonucunda doktora başvurdunuz mu? ...........Evet ........... Hayır
- Yaralanma sebebiyle işinizden yarım gün veya daha fazla uzak kaldınız mı?         ........... Evet ........... Hayır
- Yaralanmadan dolayı ne tür bir tedavi uygulandı?

..........Cerrahi ...........İlaç ...........İstirahat ..........Egzersiz

...........Kendi mesleki bilgime göre uyguladığım teknikler ( Ergonomik

koşullara ve çalışma postürüne dikkat etme, self germe v.s )

- Yaralamadan sonra klinik pratikleriniz esnasında semptomlarınızda bir artma oldu mu?         ........... Evet ........... Hayır

*Eğer* *evet ise*, ne tür aktiviteler semptomlarınızın tekrarlamasına sebep oluyor?

----------Hasta yönlendirme/transfer

----------Kaldırma

----------Uzun bir süre aynı pozisyonda kalma

----------Manuel terapi teknikleri uygulama esnasında

----------Tekrarlı hareket esnasında

----------Hasta yönlendirme/transfer

----------Baş üzerinde ki aktivitelerde

----------Uzanma

----------Merdiven çıkma

----------Çömelme

----------Yürüme

----------Uygun olmayan veya dar alanda çalışma esnasında

----------Diğer

- Geçirdiğiniz yaralanmalar iş alışkanlıklarınızın değişmesine yol açtı mı?

        ........... Evet ........... Hayır

*Eğer* *evet ise*, ne gibi bir değişiklik yaptınız?

---------Ağırlık kaldırmadan kaçınma

---------Çok sık pozisyon değiştirme

---------Çalışma süresinde değişiklik (fazla mesai,gün içine yayma v.s.)

---------Manuel teknikleri azaltma

---------Tedavi dışında hasta katılımını artırma ( ev programı gibi)

---------Hasta sorumluluğunu artırma, fzt gözlem süresini artırma

---------Mekanik alet kullanımı artırma

---------Personel kullanımı artırma

---------Semptomlar görüldüğünde veya ağrı olduğunda çalışmayı kesme

---------Gün içinde çok fazla dinlenme araları verme

---------Vücut mekaniğini düzeltme ve ergonomik koşullara uymaya çalışmak

- Yaralanma sonucu hasta ile temas süreniz limitlendi mi?

...........Evet .......... Hayır

- Olası bir yaralanmadan kaçınmak için çalışma (pratik) alanınızı sınırlandırdınız mı? ......... Evet ......... Hayır
- Bu veya olası bir mesleki yaralanmadan dolayı işinizi yada departmanınızı değiştirmeyi düşünüyor musunuz? ........... Evet ........... Hayır
